# Liquid Biopsy for EGFR Mutation Analysis in Advanced Non-Small-Cell Lung Cancer Patients: Thoughts Drawn from a Real-Life Experience

**DOI:** 10.3390/biomedicines9101299

**Published:** 2021-09-23

**Authors:** Paola Ulivi, Elisabetta Petracci, Matteo Canale, Ilaria Priano, Laura Capelli, Daniele Calistri, Elisa Chiadini, Paola Cravero, Alice Rossi, Angelo Delmonte, Lucio Crinò, Giuseppe Bronte

**Affiliations:** 1 Biosciences Laboratory, IRCCS Istituto Romagnolo per lo Studio dei Tumori (IRST) “Dino Amadori”, 47014 Meldola, Italy; paola.ulivi@irst.emr.it (P.U.); laura.capelli@irst.emr.it (L.C.); daniele.calistri@irst.emr.it (D.C.); elisa.chiadini@irst.emr.it (E.C.); 2 Biostatistics and Clinical Trials Unit, IRCCS Istituto Romagnolo per lo Studio dei Tumori (IRST) “Dino Amadori”, 47014 Meldola, Italy; elisabetta.petracci@irst.emr.it; 3 Medical Oncology Department, IRCCS Istituto Romagnolo per lo Studio dei Tumori (IRST) “Dino Amadori”, 47014 Meldola, Italy; ilaria.priano@irst.emr.it (I.P.); paola.cravero@irst.emr.it (P.C.); angelo.delmonte@irst.emr.it (A.D.); lucio.crino@irst.emr.it (L.C.); giuseppe.bronte@irst.emr.it (G.B.); 4 Radiology Unit, IRCCS Istituto Romagnolo per lo Studio dei Tumori (IRST) “Dino Amadori”, 47014 Meldola, Italy; alice.rossi@irst.emr.it

**Keywords:** non-small cell lung cancer, liquid biopsy, epidermal growth factor receptor, tyrosine kinase inhibitors

## Abstract

Background: Liquid biopsy analysis for EGFR detection in cell-free DNA (cfDNA) from NSCLC patients has become routine. The aim of this study was to explore its applicability in clinical practice. Methods: We collected data of EGFR-mutated NSCLC patients with liquid biopsy analysis. Data included test timing, concomitant tissue re-biopsy, therapy change, histology, stage, smoking habits, gender and age. All analyses were performed via a real-time PCR method to analyze EGFR mutations at exons 18, 19, 20 and 21. Variant allele frequency was performed for patients with available sequential EGFR mutation analysis in cfDNA. Overall survival was analyzed through the Kaplan–Meier method. We designed flow charts to show the real-life application of liquid biopsy. Results: We found that liquid biopsy is used in treatment-naïve patients as an alternative to EGFR detection in tumor tissue, and in patients with positive or negative EGFR from tumor biopsy. The majority of liquid biopsy analyses were performed in NSCLC patients who were disease progressive during TKI therapy. The presence of EGFR mutation in cfDNA was associated with a worse prognosis. In two patients, VAF of EGFR mutations in cfDNA was concordant with tumor volume changes. Conclusion: These findings suggest that liquid biopsy for EGFR detection can continue to be useful.

## 1. Introduction

Non-small cell lung cancer (NSCLC) represents about 85% of lung cancers, and the predominant histotype is adenocarcinoma [1]. Currently, the molecular characterization of all advanced adenocarcinoma is mandatory to identify the molecular alterations for which targeted therapies are available. Among these, mutations in the epidermal growth factor receptor (EGFR) are present in around 15% of adenocarcinomas and represent the target for first-, second- and third-generation tyrosine kinase inhibitors (TKIs), all usable in clinical practice [2,3,4,5]. The detection of EGFR mutations in tumor-tissue samples is considered the gold standard for molecular diagnostics. However, there are some circumstances where tumor-tissue analysis is very difficult for several reasons, e.g., insufficient tumor material available at diagnosis, only bone biopsy available with difficulties performing molecular analysis or patient clinical conditions may not permit invasive procedures to obtain the biological material. Moreover, patients treated with first- (Gefitinib or Erlotinib) or second-generation (Afatinib) TKIs progress with the development of EGFR T790M mutation, which represents a marker of sensitivity to treatment with the third-generation TKI Osimertinib [6]. This renders the re-characterization of the tumor as progressive disease (PD) mandatory.

In all these circumstances, liquid biopsy analysis has become a routine methodology used in clinical practice. The various laboratories dedicated to molecular diagnostics have developed and set up specific protocols for EGFR mutation analysis on cfDNA, and different analytical methods and commercial kits have been developed. As a consequence, the workflow for identifying the EGFR alterations may vary among different institutions. The aim of the present study was to describe the clinical practice use of liquid biopsy at Istituto Romagnolo per lo Studio dei Tumori “Dino Amadori” (IRST-IRCCS), highlighting the reliability of this methodology.

## 2. Materials and Methods

### 2.1. Patients

All patients with a diagnosis of advanced NSCLC who underwent a liquid biopsy for any reason at the Molecular Diagnostics Unit of Biosciences Laboratory of IRST-IRCCS between 1 January 2017 and 31 May 2019 were considered. Clinical follow-up information was collected for each patient, such as timing of liquid biopsy test, concomitant tissue re-biopsy, therapy change, histology, stage, smoking habits, gender and age. Two cases of patients with a sequential detection of EGFR mutations in liquid biopsy are described. The project was approved by the Local Ethics Committee (C.E.ROM. Protocol Number IRSTB102, Prot. 8648/2020 I.5/275, approved on 6 November 2020), and written informed consent was obtained from all patients.

### 2.2. Cell-Free DNA Analysis 

In this paper, the use of the term ‘liquid biopsy’ refers to the detection of EGFR mutations in cfDNA. Results of molecular analysis obtained on liquid biopsy were retrieved from the Molecular Diagnostics Unit data archive. Briefly, peripheral blood samples were collected in two 9 mL EDTA tubes, centrifuged at 1600× *g*, 10 min without brake. Supernatant was further centrifuged at 2000× *g*, 10 min without brake to recover plasma. cfDNA was extracted using a Maxwell^®^ RSC ccfDNA Plasma kit (Promega, WI, USA). All analyses were performed using a real-time PCR method (Real Time Easy EGFR, Diatech Pharmacogenetics, Jesi, Italy). This methodology enables the analysis of all principal mutations at exons 18, 19, 20 and 21 of the EGFR gene. For the two patients for whom a sequential EGFR mutation analysis was performed, the analysis was also performed using the Myriapod NGS Cancer panel DNA (Diatech Pharmacogenetics, Jesi, Italy), with the aim of assessing the variant allele frequency (VAF) of gene mutations, defined as the number of variant reads divided by the number of total reads, reported as a percentage. 

### 2.3. Statistical Analyses

Data were summarized using mean ± standard deviation, median and minimum and maximum values or interquartile range (IQR), as appropriate, for continuous variables and by means of frequencies and percentages for categorical ones. Overall survival (OS) was reported by Kaplan–Meier curves, and curves were compared by means of the log-rank test. Hazard ratios (HRs) and 95% confidence intervals (CIs) were obtained by applying the Cox proportional hazards model. The presence of confounding was investigated looking at the association between the potential confounder and the main exposure of interest, as well as between the potential confounder and OS. Flow charts were designed to show the real-life application of recommendations from IASLC on liquid biopsy for advanced NSCLC [7]. Analyses were performed using R statistical software version 3.6.2.

## 3. Results

From January 2017 to May 2019, a total of 221 liquid biopsy samples derived from 137 patients were analyzed at the Molecular Diagnostics Laboratory of IRST-IRCCS for EGFR molecular testing. Patients’ characteristics are summarized in Table 1. The majority of patients were female (63.5%), with a median age of 69 years [IQR: 16.4], non-smokers (50.9%), had an adenocarcinoma (86.3%) and a stage IV tumor (89.6%), Table 1.

Of the 221 liquid biopsy samples, EGFR-activating mutations were investigated in 215 samples; such mutations were not analyzed for six patients. Of these, 135 (62.8%) were EGFR wt. Moreover, 41 (19.1%) showed an exon 19 deletion, 28 (13.0%) an L858R mutation, 4 (1.9%) an exon 18 mutation. In total, 96 (70.1%) patients underwent a single liquid biopsy test, 23 (16.8%) underwent two tests, 6 (4.4%) underwent three tests, 7 (5.1%) underwent four tests, and 5 patients (3.6%) underwent more than 5 tests.

For 26 patients (19.0%), the liquid biopsy test was performed without knowledge of the EGFR status in the tumor tissue. Of these, only one patient showed an EGFR mutation in liquid biopsy, an L858R mutation. Ninety-two (67.2%) patients had an EGFR mutation detected in tumor tissue (52 exon 19 deletion, 33 L858R, 3 exon 18 mutations and 4 exon 20 mutations). In these patients, EGFR mutation was found in 85.9% (4 in exon 18, 41 patients had an exon 19 del, 1 an exon 20 insertion, 27 an L858R mutation and 6 an L861Q mutation). Moreover, 19 (13.9%) patients had wt EGFR detected in tumor tissue, and all tested wild-type in liquid biopsy.

Of all liquid biopsy analyses performed on patients receiving at least one therapeutic regimen, 48 (23.0%) were requested at baseline, 132 (63.1%) during treatment with a TKI and 29 (13.1%) during treatment with other therapies (for 12 patients, such information was missing).

Overall, T790M mutation in liquid biopsy was found in 22 (10.0%) of all analyzed samples: 1 sample showed the T790M mutation at baseline, 14 during first-line TKI therapy, 1 during second-line therapy with TKI and 2 during treatment with other agents. In four cases, this information was missing. Of the 22 cases, 16 (72.7%) samples also showed an exon 19 del, whereas 6 (27.3%) cases showed an L858R mutation.

At disease progression with TKI therapy, T790M mutation was found in 62 cases (39.2% of the cases for which this information was available).

Tumor re-biopsy was performed in 63 patients. For 47 of these, EGFR mutation analysis was performed, revealing the presence of activating mutation in 35 (74.5%) cases. T790M was investigated in 37 samples, and was found in 11 (29.7%) cases.

Overall, a therapy change after the result of liquid biopsy analysis was observed in 42 (24.9%) cases; in 52 cases, this information was not available.

**Figure 1:**
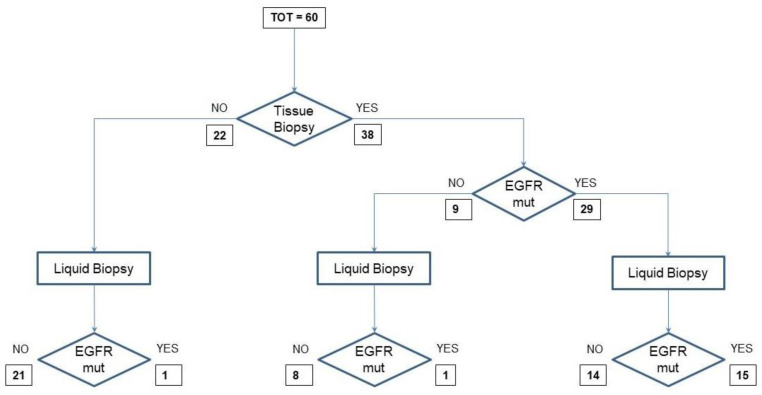
Samples from treatment-naïve patients and flow-chart of routine EGFR mutation analysis.

To illustrate the allocation of each liquid biopsy analysis in the context of the various possibilities of its use in clinical practice, we designed a flow chart for samples from treatment-naïve patients and another one for samples from disease-progressive patients during TKI therapy. In the first flow chart are 60 samples from treatment-naïve patients with a diagnosis of NSCLC. Twenty-two of them could not undergo EGFR analysis in tumor tissue because of inadequate samples. They were analyzed for EGFR mutation in cfDNA; however, only one sample was positive for the exon 21 L858R EGFR mutation. The 38 samples from patients tested in tumor tissue for EGFR-activating mutations included 29 samples from EGFR-mutant and 9 from EGFR wild-type patients. Among the 29 samples, 14 tested negative and 15 positive for EGFR mutations in cfDNA. The remaining nine samples included eight negative and one positive for EGFR mutations in cfDNA (Figure 1).

**Figure 2:**
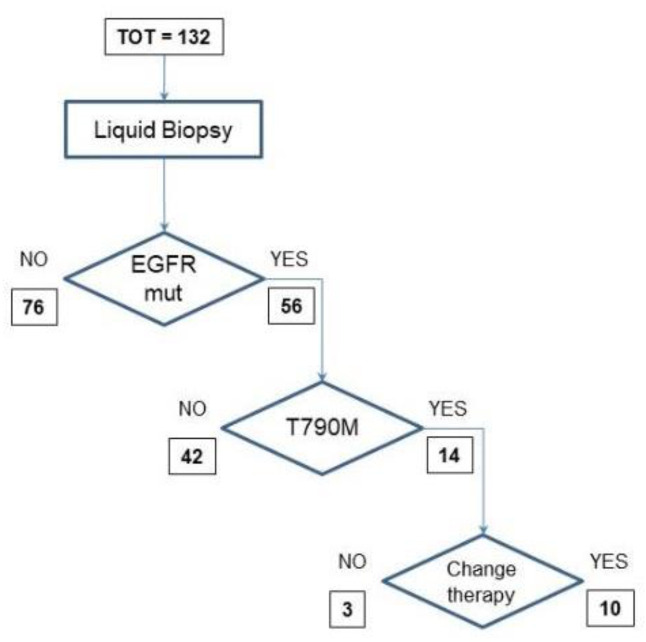
Flow-chart for EGFR mutation analysis for patients in progressive disease after TKI treatment.

There were 132 blood samples from NSCLC disease-progressive patients during TKI therapy. Seventy-six were negative for EGFR mutations in cfDNA. The other 56 were positive for activating EGFR mutations, including 14 samples positive for the exon 20 T790M EGFR mutation. This positive result for the resistance mutation led to change in therapy in 10 cases (Figure 2).

**Figure 3:**
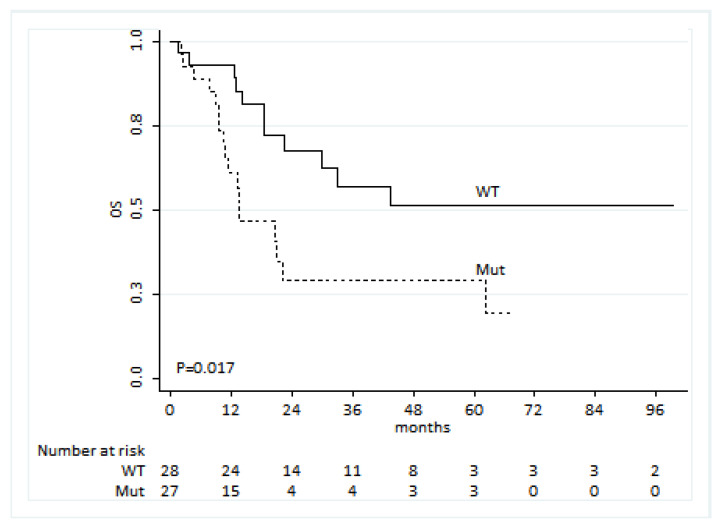
Overall survival (OS) of patients by EGFR mutation in cfDNA.

We identified 60 patients showing an activating EGFR mutation at baseline and receiving a first-line treatment with a TKI. For 55 of these patients, survival data were available, and we observed that patients showing the presence of an EGFR mutation in liquid biopsy were characterized by a worse OS compared to those for which no EGFR mutation was evident (HR: 2.56, 95% CI: 1.18–5.53, *p* = 0.017, Figure 3). Among the demographic and clinical covariates, smoking was the only one associated with OS. However, this factor was not associated with the presence of an EGFR mutation. Thus, no confounding seemed to be present. Including both factors in the Cox model, EGFR-mutated patients reported more than double the risk of death than wt patients (HR: 2.24, 95% CI: 1.02–4.92, *p* = 0.044), whereas current smokers had a three-fold increased risk over never smokers (HR: 3.04, 95% CI: 1.06–8.73, *p* = 0.039 and HR: 0.96, 95% CI: 0.41–2.23, *p* = 0.923 for current vs. never smokers and for ex- vs. never smokers, respectively).

Blood samples from two patients were analyzed for EGFR mutations in cfDNA for at least three time points. We describe these two cases and report the graphs with imaging and cfDNA analyses in terms of variant allele frequency (VAF).

Case presentation 1

**Figure 4:**
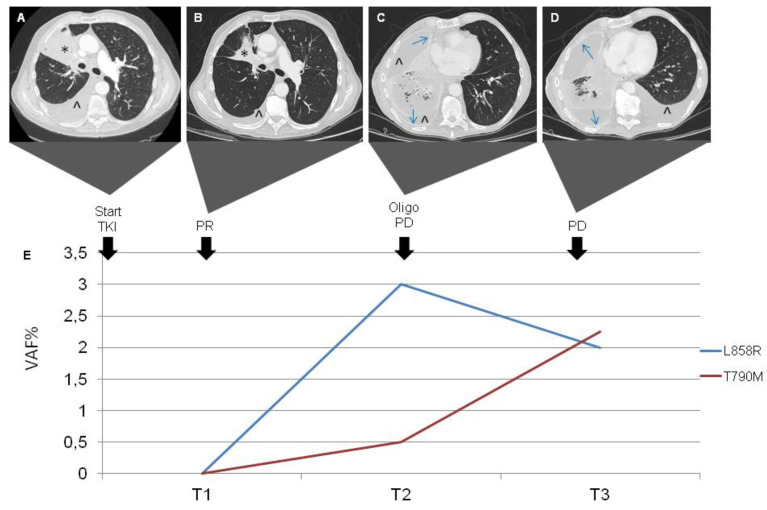
History of disease of the patient described as Case 1. Radiologic responses to TKI treatment paired with circulating activating and resistance EGFR mutation. CT shows reduction in size both of lung cancer (*) and pleural effusion (^) localized in the right lung from baseline (**A**) to the fist control (**B**). Relapse with complete filling of right pulmonary air spaces, malignant pleural thickness (blu arrow) and pleural effusion (**C**,**D**). Longitudinal VAF of L858R and T790M mutations in cfDNA (**E**).

In April 2018, a 78-year-old Caucasian woman with no smoking history came to our attention because of persistent dry cough. A total body computed tomography (CT) evidenced a primary lung carcinoma with lymphangitis, minimal pericardial effusion, ilo-mediastinal lymph nodes and multiple bone metastases. The stage was IVb (cT3N3M1c) according to the UICC 8th edition, and the Eastern Cooperative Oncology Group performance status (ECOG PS) was 1. The fibrobronchoscopy (FBS) with lymph node biopsy diagnosed a lung adenocarcinoma. Immunohistochemistry was performed and revealed: ALK negative, ROS1 negative, PD-L1 negative. The first next-generation sequencing (NGS) analysis on cells isolated from cytological preparations was performed in May 2018 and evidenced EGFR Del19 mutation (Glu746_Ala750del). Based on this finding, Gefitinib (250 mg per day) was started in May 2018. A subsequent 18F-FDG PET/CT scan in July 2018 (T1) described a complete response (CR). Consequently, Gefitinib was continued and another cfDNA analysis by NGS was performed, revealing neither exon 19 deletion nor exon 20 EGFR mutations. The following cfDNA NGS analysis in September 2018 (T2) identified EGFR 19 deletion without exon 20 mutation, while a 18F-FDG PET/CT scan in October 2018 confirmed the CR. Gefitinib was discontinued in January 2019 (T3) due to radiological progressive disease (PD) of lung, lymph nodes, bone and brain. The concomitant NGS analysis on cfDNA reported the appearance of a T790M mutation and an increase in exon 19 deletion, suggesting resistance to Gefitinib (Figure 4).

The patient thereby received Osimertinib (80 mg per day) for two months with a subsequent decline in physical conditions that led to discontinuing Osimertinib in April 2019 and death in May 2019. For the entire duration of the treatment, Gefitinib was well tolerated without adverse effects.

Case presentation 2

**Figure 5:**
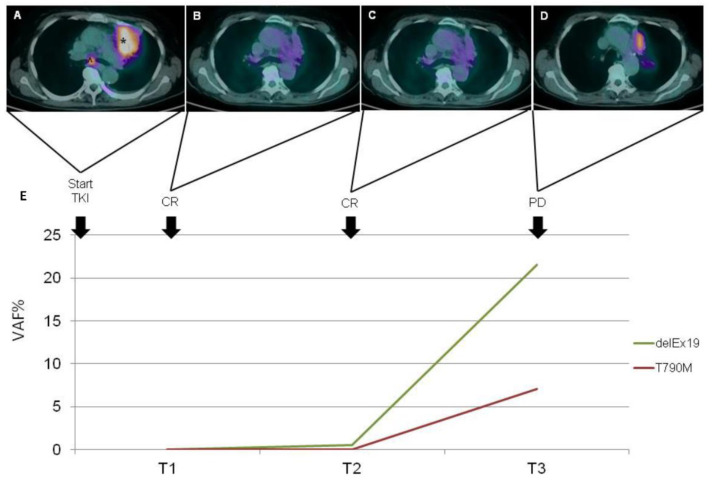
History of disease of the patient described as Case 2. Radiologic responses to TKI treatment paired with circulating activating and resistance EGFR mutation. PET-CT Images show size reduction without FDG-uptake, consisting with complete response, of the lung cancer localized in the left upper lobe (*) and adenopathy at subcarinal level (˄) from baseline (**A**) to controls at 3 and 6 months (**B**,**C**); PET-CT in the fourth image shows a relapse of tumor (**D**). Longitudinal VAF of delEx19 and T790M mutations in cfDNA (**E**).

In December 2016, a 72-year-old man with a previous smoking history (40 p/y) was referred to our center complaining of cough and dyspnea. A total body CT scan evidenced a lung neoplasm with metastases in bone and lymph nodes. The diagnosis of adenocarcinoma was confirmed by FBS with a lung biopsy. The stage was IVb (cT4N3M1c), and the ECOG performance status was 2. The NGS analysis on cells isolated from cytological preparations found EGFR exon 21 mutation L858R and, due to this finding, Afatinib (40 mg per day) was prescribed with an initial radiological partial response (PR) in March 2017 (T1), associated with a negative cfDNA analysis for both exon 21 L858R and exon 20 T790M mutations. In June 2017, a PD was observed only in the lung (oligoPD). Afatinib was then continued at a dosage of 30 mg per day due to gastrointestinal toxicity (persistent diarrhea G1). In July 2017, a 18F-FDG PET/CT scan was consistent in PR. A CT scan in October 2017 demonstrated further pulmonary response with stability of the other sites. He continued Afatinib, developing a cutaneous G2 toxicity (skin rash, perionypsis, itch, palmar fissures) treated with Doxycycline. In December 2017, he developed worsening dyspnea with consequent evacuative thoracentesis (2500 cc drained) and positioning of pleural drainage complicated by hydropneumothorax. In January 2018 (T2), he underwent pleural talc, and the drainage was removed. He also developed pneumonia treated with multiple antibiotic lines and steroids. Afatinib was temporarily suspended for that reason and because of diarrhea and folliculitis. Through NGS-based cfDNA analysis performed at that time, we found that the EGFR L858R mutation of exon 21 was not associated with T790M. Based on radiological stable disease (SD) and resolution of adverse effects, Afatinib was resumed in February 2018. In April 2018 (T3), the last NGS sequencing of a peripheral blood sample revealed L858R on exon 21 as well as T790M on exon 20, concomitant with radiological PD at CT scan and clinical worsening (Figure 5). Death occurred in the same month.

## 4. Discussion

In NSCLC treatment-naïve patients, liquid biopsy could represent an advantage for molecular diagnostics, as a patient’s tissue could be reserved for PD-L1 immunohistochemistry. On the other hand, not all tumors release a sufficient amount of DNA for mutational assessment in the bloodstream; in advanced-stage disease, an approximate sensitivity of 85% is the highest reached. Moreover, newly diagnosed patients with slow-growing tumors are more frequently associated with false-negative results in plasma mutation analysis compared to patients with more disseminated disease [8]. Variant allele frequency (VAF) in plasma can dramatically change because of therapy. For this reason, cfDNA analysis should be performed before any line of therapy, as an actionable mutation in a patient could become undetectable within one or two weeks of treatment [9]. Treatment-naïve patient selection for EGFR mutation assessment on cfDNA should take into account the same criteria as for tumor tissue: advanced stage or metastatic nonsquamous NSCLC, squamous NSCLC in younger patients and/or never smokers. Liquid biopsy is drawn by a minimally invasive practice and is possible at diagnosis in all patients for EGFR mutations. It is also recommended when tumor tissue is poor or unavailable. Moreover, analysis on liquid biopsy becomes the best feasible diagnostic tool when EGFR detection in tumor tissue takes more than 2 weeks, or tissue biopsy may be risky or contraindicated. Bone biopsies represent a particular condition, as they are useful for histological diagnosis, but decalcification procedures may damage nucleic acids. While the detection of an actionable mutation in cfDNA is a necessary and sufficient condition for beginning a targeted therapy, a negative result should be addressed with a secondary analysis [7]. For ALK rearrangement detection in treatment-naïve patients’ cfDNA, prospective studies are needed to mark its reliability, as retrospective studies highlighted that qPCR is not the best tool to detect this alteration. Droplet digital PCR (ddPCR) has shown interesting results; however, this approach still requires technical validation. On the other hand, NGS could provide an acceptable sensitivity and an optimal specificity; however, the data collected to date are not specific for ALK rearrangement [10,11,12]. The assessment of T790M mutation in EGFR exon 20 after resistance to first- and second-generation EGFR-TKIs allows the administration of the third-generation EGFR-TKI, Osimertinib [13]; however, the use of Osimertinib as gold standard in first-line treatment, on the basis of the results from the FLAURA trial, changed this scenario [5]. However, liquid biopsy may be a useful tool to identify resistance mechanisms to Osimertinib, and the assessment of EGFR alterations should be performed for patients facing clinical or radiological progression disease during first- or second-generation EGFR TKI treatment. If a plasma sample from a progressive patient is wt for T790M mutation, a further assessment with a more sensitive molecular methodology, or the molecular evaluation of DNA from a tumor re-biopsy should be performed. In fact, the qPCR methodology demonstrated a low sensitivity across different clinical trials, ranging from 46% to 65.7% [14]. In our case series, qPCR reached a sensitivity of 51.7% (15/29), confirming that a negative result using this methodology could be a false negative, and that a more sensitive technology should be applied. Alternatively, a more comprehensive analysis on cfDNA could be considered, e.g., using an NGS panel able to detect a wide spectrum of genomic alterations added to T790M resistance mutation. As for the assessment of actionable mutations, a positive result for EGFR T790M is sufficient to administer Osimertinib as second-line treatment after treatment failure of a first- or second-generation EGFR-TKI. It has to be highlighted that EGFR T790M is not the only mechanism of resistance to first- and second-generation EGFR-TKIs, and it could not be detected even in a tumor re-biopsy. In this case, NGS analysis may be useful to track a mutational profile of a progressive tumor and may help to find new resistance mechanisms, useful to treatment choice and in directing patients to a clinical trial or an expanded access program. If the actionable (primary) EGFR mutation is detected, the absence of the T790M mutation is more reliable, while if none of the two mutations is detected, it could be assumed that the tumor is not releasing a sufficient amount of DNA in the bloodstream; thus, liquid biopsy analysis could be more reliable later during treatment [9].

Previously, other researchers explored the role of liquid biopsy practice for EGFR mutation testing in NSCLC patients as a real-life experience. Wei et al. compared liquid biopsy with tissue re-biopsy in 375 EGFR-mutant NSCLC patients. They found around 63% sensitivity and 83% specificity using a ddPCR assay for plasma samples. However, in their institution liquid biopsy was usually performed when tissue re-biopsy was not indicated because of clinical limitations [15]. In the paper by Soria-Comes et al., around 87% among 89 eligible patients had a result from liquid biopsy consistent with that from tissue biopsy. These authors achieved around 70% sensitivity and 92% specificity [16]. Another recent real-world analysis of T790M mutation in cfDNA found a significantly higher positivity of T790M mutation in those patients with baseline L858R mutation [17]. These results are consistent with those reported by other authors [18,19,20]. Li et al. also found significantly higher T790M positivity in patients experiencing a local progression than in those with gradual or dramatic progression. Conversely, they did not observe any relationships of T790M mutation in cfDNA with age, sex or type of EGFR-TKI [17]. In our paper, sensitivity and specificity were not the main purposes of this work. However, we gathered all the analyses for EGFR mutations in blood samples from NSCLC patients. These analyses were made in clinical practice in various settings. Thus, we represented all the analyses in two different flow charts, one for samples from treatment-naïve patients and one for patients with progressive disease after first- or second-line EGFR TKI treatment. From these charts, some thoughts emerge. Liquid biopsy for the detection of activating EGFR mutations can be helpful when tissue biopsy is not feasible. When EGFR is wild-type in tumor tissue, liquid biopsy can help to identify around 10% of EGFR-positive patients (one out of nine in this case series). Conversely, in those patients with an EGFR mutation detected via tumor biopsy, this result was confirmed in around 50% of cases. This apparent scant sensitivity is possibly due to the method of real-time PCR we used in clinical practice in that period. Perhaps, the use of ddPCR is more appropriate for confirmation of the results from tumor biopsy, given that this different technique can reach sensitivity higher than 80%. In fact, in a work by Wei et al., they used ddPCR, and NGS for validation, and reached a higher sensitivity than in our work [15]. Similarly, Soria-Comes et al. achieved a 70% sensitivity via qPCR [16]. Finally, Li et al. used amplification refractory mutation system (ARMS); however, they tested only T790M mutation of EGFR in cfDNA, and these data in tissue samples are not available in this work [17]. For these reasons, our results are not fully comparable to those reported in the other real-life experiences. From our analyses and the other cited works, the main purpose, for the use of cfDNA for EGFR detection during treatment with first- or second-line EGFR TKI, seems to be the identification of T790M EGFR resistance mutation to address the change of therapy with Osimertinib. However, this option will naturally involve fewer patients in near future because, recently, upfront Osimertinib became the standard treatment for EGFR-positive patients with advanced NSCLC. Finally, the two cases for which serial data of EGFR mutations in cfDNA during TKI were available support the possibility of using this method to follow the evolution of this malignancy under treatment and combine these results with imaging. This application can also have a prognostic role, as the presence of EGFR mutations in cfDNA is associated with worse OS. Perhaps, EGFR mutations can be detectable in cfDNA when the tumor load is higher. In the work by Wei et al., they found the onset of T790M EGFR mutation during upfront TKI treatment and of C797S resistance EGFR mutation during second-line Osimertinib in liquid biopsy from four patients [15].

The therapeutic scenario for oncogene-addicted advanced NSCLC patients is rapidly changing. Consequently, the detection of a specific resistance mutation will not be sufficient to personalize subsequent treatment after the progression under a first-line TKI. For this reason, high hopes are placed in the use of next-generation sequencing (NGS) to screen for multiple genetic alterations both in tissue and in plasma. However, the validity of this method in comparison with standard methods has yet to be defined.

## 5. Conclusions

In this work, we evaluated the applicability of liquid biopsy analysis for EGFR detection in EGFR-mutated NSCLC patients. We collected data on the timing and results of these analyses. We found that it was used in treatment-naïve patients as an alternative to EGFR detection in tumor tissue, when this was not feasible, and also in patients with positive or negative results of EGFR detection in tumor tissue. However, the majority of liquid biopsy analyses were performed in NSCLC patients who were disease-progressive during TKI. In this case, EGFR T790M was detected and, when the result of this analysis was positive, the majority of patients changed treatment with TKI to Osimertinib. The presence of EGFR mutation in ctDNA was associated with a worse prognosis. We showed in two patients that VAF of EGFR mutations in ctDNA is concordant with tumor volume changes. These results suggest that liquid biopsy can continue to be useful, even though Osimertinib is currently mainly used as first-line treatment in patients with an activating EGFR mutation.

## Figures and Tables

**Table 1:** Clinico-pathological patient characteristics (*n* = 137).

	*n*	%
**Gender**		
F	87	63.5
M	50	36.5
Age at diagnosis (yrs)		
median [min-max]	68.6 [34.1–91.8]	
missing	20	
**Smoking habit**		
Non-smoker	55	50.9
Current smoker	11	10.2
Former smoker	42	38.9
missing	29	
**Histotype**		
Adenocarcinoma	102	86.3
Squamous carcinoma	3	2.6
Other	13	11.1
missing	20	
**Stage**		
IIIA	5	4.3
IIIB	7	6.1
IV	103	89.6
missing	22	

## Data Availability

Data are available by the corresponding author upon reasonable request.
